# Essential role of the metabolite α-ketoglutarate in bone tissue and bone-related diseases: Role of α-ketoglutarate in bone tissue and bone-related diseases

**DOI:** 10.3724/abbs.2025020

**Published:** 2025-02-19

**Authors:** Zuping Wu, Yuzhe Guan, Qian Chen, Ruifeng Song, Jing Xie, Xin Zhang, Yan Wang, Qianming Chen, Xiaoyan Chen

**Affiliations:** 1 Stomatology Hospital School of Stomatology Zhejiang University School of Medicine Zhejiang Provincial Clinical Research Center for Oral Diseases Key Laboratory of Oral Biomedical Research of Zhejiang Province Cancer Center of Zhejiang University Engineering Research Center of Oral Biomaterials and Devices of Zhejiang Province Hangzhou 310000 China; 2 State Key Laboratory of Oral Diseases West China Hospital of Stomatology Sichuan University Chengdu 610064 China; 3 Shandong University & Shandong Key Laboratory of Oral Tissue Regeneration & Shandong Engineering Research Center of Dental Materials and Oral Tissue Regeneration & Shandong Provincial Clinical Research Center for Oral Diseases Jinan 250000 China; 4 ShengSi County People’s Hospital Zhoushan 316000 China

**Keywords:** α-ketoglutarate, bone metabolism, osteoporosis, osteoarthritis, rheumatoid arthritis, periodontitis

## Abstract

Bone metabolism in bone tissue is constantly maintained in a state of dynamic equilibrium. The mass of bone and joint tissues is determined by both bone formation and bone resorption. It is hypothesized that disrupted metabolic balance leads to osteoporosis, osteoarthritis, rheumatoid arthritis, and bone tumors. Such disruptions often manifest as either a reduction or abnormality in bone mass and are frequently accompanied by pathological changes such as inflammation, fractures, and pain. α-Ketoglutarate (α-KG) serves as a pivotal intermediate in various metabolic pathways in mammals, significantly contributing to cellular energy metabolism, amino acid metabolism, and other physiological processes. α-KG may be a therapeutic target for a variety of bone-related diseases, such as osteoporosis, osteoarthritis, and rheumatoid arthritis, because of its role in maintaining the metabolic balance of bone. After the application of α-KG, bone loss and inflammation in bone tissue are alleviated. This review focuses on the regulatory effects of α-KG on various cells in bone and joint tissues. Owing to the regulatory effect of α-KG on the balance of bone metabolism, the application of α-KG in the treatment of osteoporosis, osteoarthritis, rheumatoid arthritis, bone tumors, and other bone tissue diseases has been clarified.

## Introduction

As an intermediate in a variety of metabolic processes, α-ketoglutarate (α-KG) is also known as 2-oxoglutarate and is closely involved in the energy metabolism process represented by carbohydrate metabolism in the body [1]. Carbohydrate metabolism depends on the energy supply of the tricarboxylic acid cycle (TCA), which is the main pathway for acetyl-CoA to completely decompose into water and carbon dioxide and generate energy, and it is also the precursor source of much biosynthesis [2]. α-KG, as a rate-determining intermediate, is crucial in cell energy metabolism because it connects intracellular carbon and nitrogen metabolism between isocitrate and succinyl coenzyme A. Additionally, α-KG is closely involved in the amino acid cycle [3]. As a precursor of amino acids such as glutamine and glutamic acid, α-KG plays a direct role in energy production and a wide range of cellular chemical reactions. α-KG provides an energy source, stimulating protein synthesis, inhibiting protein degradation in muscle, and serving as a significant metabolic fuel for gastrointestinal cells [2]. In the intestine, proline, leucine, and other amino acids are formed by α-KG in the intestinal epithelium [4]. Moreover, studies have shown that the synthesis of ketoglutarate reductive amination around blood vessels is also an important source of glutamic acid in bone tissue [5].

The dynamic balance between mature osteoblast activity and osteoclast activity involves complex processes affecting bone metabolism [ 6, 7]. Various bone-related diseases are often accompanied by a loss of balance between osteoblast and osteoclast activity, as well as inflammatory lesions in cartilage and synovial tissue. Furthermore, the proliferation, differentiation and apoptosis of various cells in bone tissue are closely related to energy metabolism [8]. The activation of mitochondrial physiological activities and the metabolic transition to oxidative phosphorylation are involved in the differentiation, proliferation, and maturation of mesenchymal stem cells (MSCs) [9]. In one recent study, the levels of mitochondrial biogenesis-related proteins were found to be elevated, along with increased levels of tricarboxylate cyclase and respiratory chain complex subunits. This resulted in an increase in oxygen consumption, the mitochondrial membrane potential, and the intracellular ATP content, indicating that the oxidative metabolism of mitochondria is enhanced during bone metabolism [10]. The effect of α-KG on bone tissue is affected by a variety of mechanisms. The production and utilization of α-KG not only changes the bone metabolism balance but also affects bone mass. α-KG also affects the proliferation and differentiation of various cells in bone tissue by promoting osteogenesis, inhibiting osteoclast differentiation, and enhancing the proliferation of chondrocytes and synoviocytes. Researchers have also reported that α-KG promotes demethylation and histone modification in stem cells to induce differentiation [ 11– 13]. A change in the α-KG concentration leads to epigenetic changes, including DNA methylation and demethylation, which affect bone cell metabolism and produce related physiological and pathological effects [14]. In addition, α-KG can change bone metabolism by affecting the endocrine system. This review focuses on the role of α-KG in bone metabolism and bone-related diseases from several angles.

## α-KG

**Figure 1:**
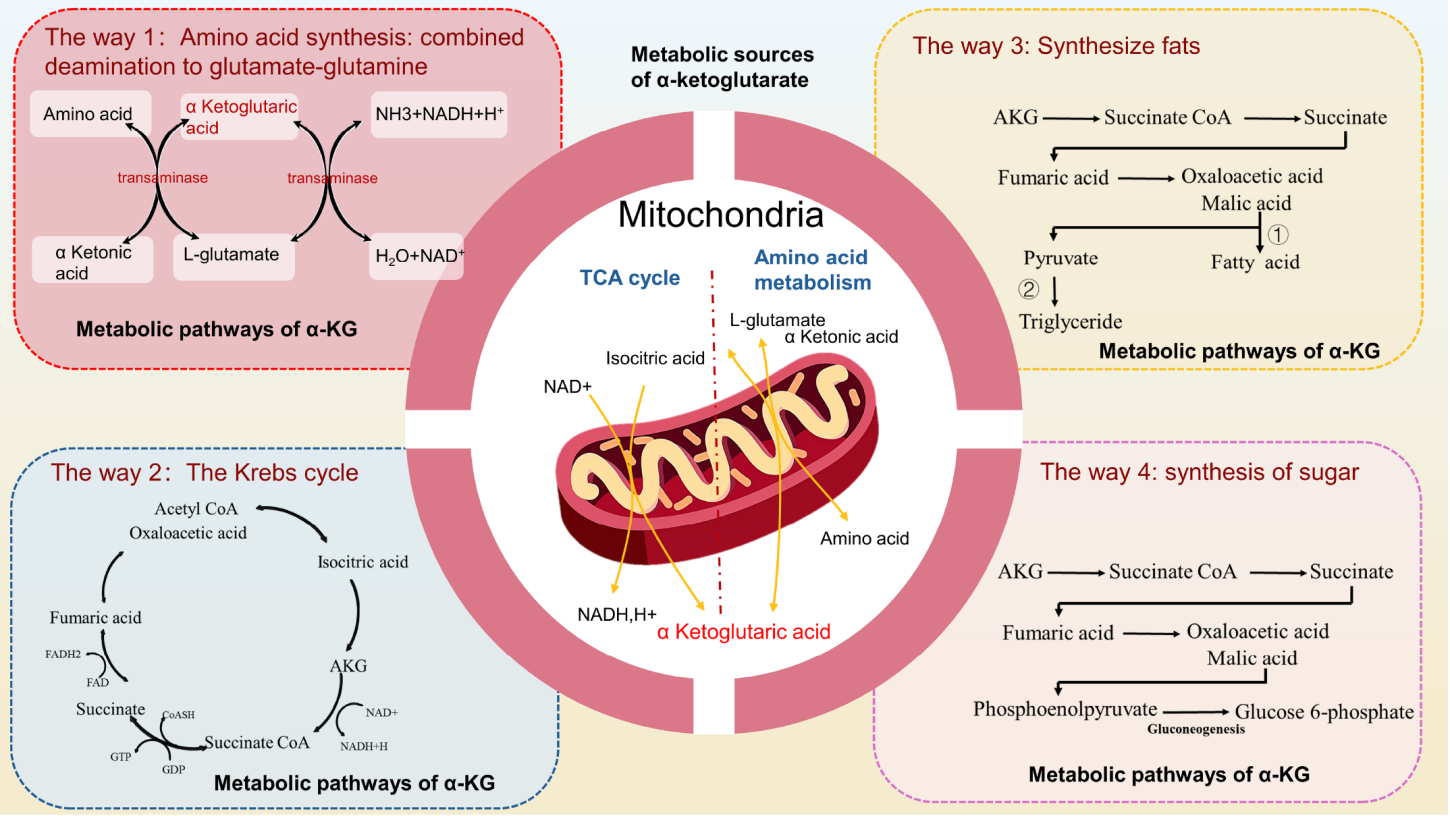
Figure 1 Sources and metabolism of α-ketoglutarate Metabolic sources of α-ketoglutarate: TCA cycle production or the amino acid metabolic pathway. Metabolic pathways of α-KG: (1) Synthetic amino acid: combined deamination to produce glutamic acid glutamine (2) Energy supply for cyclic oxidation of TCAs. (3) Fat synthesis (acetyl coenzyme A forms fatty acids through the TCA cycle, or pyruvate is generated through gluconeogenesis, and pyruvate is converted into glycerol through glycolysis). (4) Synthetic sugars (through the TCA cycle, pyruvate is generated from oxaloacetic acid, and glucose is synthesized through gluconeogenesis)

In cells, α-KG is associated with carbon-nitrogen metabolism and is a metabolic intermediate in the TCA cycle. When carbon source substances are transported into cells, they undergo glycolysis to form pyruvic acid (PYR), which in turn forms α-KG under the action of the pyruvic dehydrogenase complex (PDHC), citrate synthase, aconite, and isocitrate dehydrogenase [1]. Under the action of α-KG dehydrogenase, α-KG is further metabolized into succinyl coenzyme A (COA) and then enters the next carbon metabolism node. As a result of this process, cell growth and proliferation are supported by carbon source materials and energy [15]. In addition to regulating metabolism, α-KG also has many other physiological functions, such as promoting autophagy, alleviating inflammation, delaying aging, and prolonging life [ 16– 19] ( Figure 1).

### Structure, properties, and metabolic pathway of α-KG

α-KG can be decarboxylated to succinic acid, water, and carbon dioxide under the action of α-KG dehydrogenase and is ultimately oxidized and decomposed completely through the TCA cycle. α-KG participates in the amino acid metabolic pathway, reacts with ammonia under the action of glutamate dehydrogenase, converts it into glutamate, and further generates glutamine and glutathione. α-KG can also produce glucose molecules through gluconeogenesis and glycolysis. Glycerol and fatty acids can also be produced from α-KG via the fatty acid metabolic pathway [20] ( Figure 1).

### Function of α-KG

#### α-KG participates in the carbon cycle and energy metabolism

α-KG participates directly in the oxidative energy supply of the body through the TCA cycle. It can dehydrogenate to succinyl CoA and nicotinamide adenine dinucleotide (NADH), accompanied by the release of a large amount of energy. On the other hand, it can also promote the catabolism of fatty acids and provide energy for the body. Under normal physiological conditions, free fatty acids are nutrients for the energy supply, and long-chain fatty acid β-oxidation can produce a large amount of ATP [1]. After they absorb exogenous α-KG, intestinal villus epithelial cells replenish a large amount of energy for the body through the carbon cycle. Moreover, α-KG also participates in the synthesis of type I collagen and amino acid metabolism. Intestinal absorption is an important way for the body to obtain α-KG and energy substances [21].

#### α-KG is involved in amino acid metabolism

Glutamine can be produced by glutamine synthase via ATP. Glutamine is the substrate of many physiological processes. Glutamic acid and glutamine are the main energy sources of intestinal mucosal cells, and α-KG, as the precursor to these two amino acids, can be converted into glutamic acid and glutamine in the gut to provide energy [22]. Studies have shown that adding α-KG to the diet of rats can effectively increase the depth of crypts to the height of the villus, improve the morphology of the small intestinal mucosa, and enhance the absorption and barrier function of the small intestinal mucosa [23].

#### α-KG serves as a nitrogen transporter

Another function of α-KG is to bind with the nitrogen formed in cells, thus preventing excessive accumulation of nitrogen in cells. Amino acids are bound to α-KG through transamination and enter the urea cycle as the blood is transferred to the liver. During the urea cycle, glutamine receives the amino group obtained by transamination and forms the excitatory neurotransmitter glutamate to complete amino transport [24].

#### The special role of α-KG in bone tissue

α-KG plays a role in regulating physiological activities in human tissues and systems. In the cardiovascular system, α-KG plays an active role in regulating lipid metabolism [25]. In addition, supplementation with α-KG can significantly improve the cardiac index. In addition, α-KG has immune-enhancing properties and can maintain the intestinal barrier [26]. Many studies in the field of bone tissue research have shown that α-KG can regulate the level of bone metabolism and promote the level of osteogenesis [27]. The application of α-KG in animal feed can effectively improve bone mineral density and inhibit female osteoporosis [ 28, 29].

We discuss the regulatory effects of α-KG on bone tissue cells and their applications in various diseases related to bone.

## Regulation of Various Cells in Bone Tissue by α-KG

### Mesenchymal stem cell differentiation is regulated by the α-KG content

**Figure 2:**
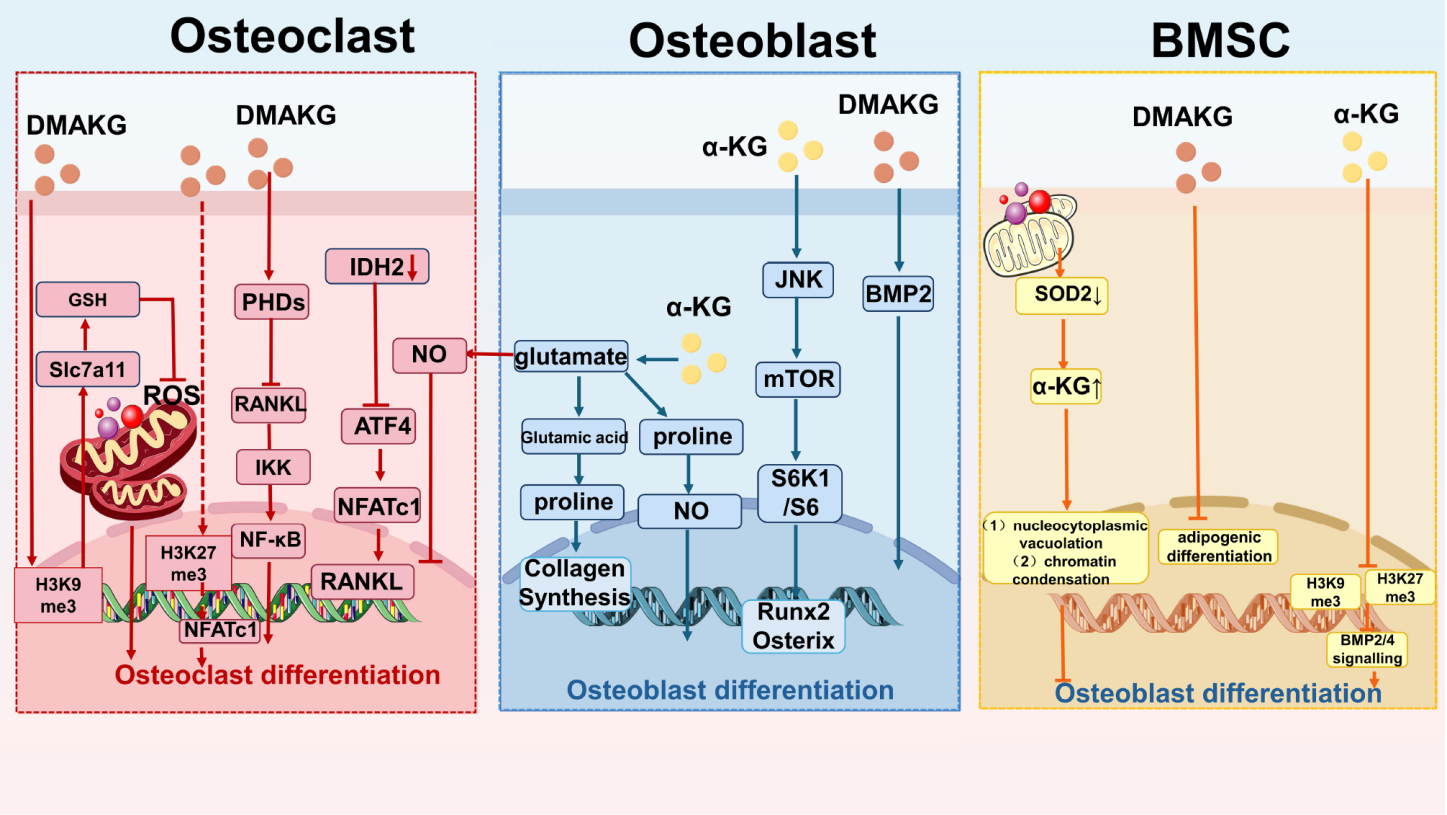
Figure 2 Regulation of α-KG in osteoblasts, osteoclasts, and mesenchymal stem cells In osteoblasts, α-KG promotes the secretion of glutamate, proline and collagen. In addition, α-KG activates the JNK pathway and downstream of mTOR/S6K1/S6, promotes the expression of Runx2 and Osterix, and then promotes osteogenesis. The α-KG analogue DMAKG activates the BMP2 pathway and promotes osteoblast maturation and bone mineralization. Additionally, α-KG participates in the metabolism of glutamate and arginine through IDH, promotes the secretion of NO by osteoblasts, and increases the activity of osteoblasts. In osteoclasts, α-KG inhibits ROS accumulation in mitochondria through the glutathione pathway, alleviates oxidative stress, and inhibits osteoclast differentiation and maturation. In osteoblasts, α-KG is converted to glutamine, and arginine is subsequently produced to release NO, which inhibits osteoclast maturation through a paracrine mechanism. In IDH2-deficient individuals, the ATF4-NFATc1 pathway in osteoblasts is inhibited, resulting in insufficient secretion of RANKL, which subsequently affects osteoblasts to promote their differentiation and maturation. DMAKG promotes H3K27me3 demethylation inhibition and inhibits osteoclast maturation through the NFATc1 pathway. DMAKG promotes H3K9me3 demethylation, activates the expression of SLC7A11, produces GSH, clears ROS accumulated in mitochondria, and alleviates oxidative stress. Mesenchymal stem cells. (1) Increased glycolysis in stem cells inhibits the expression of superoxide dismutase 2 (SOD2) in cells with excessive accumulation of α-KG, leading to vacuolation of the nuclear cytoplasm and chromatin condensation of mesenchymal precursors, which inhibits the activity of stem cells. (2) DMAKG inhibits the adipogenic differentiation of mesenchymal stem cells and promotes the osteogenic differentiation of stem cells. 3. α-KG can inhibit the accumulation of H3K9me3 and H3K27me3, upregulate BMP signaling, and promote osteogenic differentiation and osteoblast maturation.

MSCs can be differentiated in three main directions: osteogenesis, adipogenesis, and chondrogenesis. α-KG can affect gene transcription through the regulation of energy metabolism and epigenetic modifications to regulate the proliferation and differentiation of MSCs. Changes in the production and utilization of α-KG affect the performance of stem cells to a certain extent. In the field of energy metabolism research, mitochondria are organelles in which α-KG participates in energy metabolism. When mitochondrial function changes, oxidative stress usually occurs. Singh *et al*. [20] reported that the deficiency of superoxide dismutase 2 (SOD2) function and the impairment of pyruvate and L-glutamine metabolism in mesenchymal precursor cells with mitochondrial dysfunction lead to an increase in superoxide anion free radicals and excessive accumulation of α-KG in mesenchymal precursor cells. Excessive α-KG causes nucleocytoplasmic vacuolation and chromatin condensation in mesenchymal precursor cells by increasing DNA damage and inhibiting the differentiation potential of these cells [20] ( Figure 2). The results of Wang’s study indicated that the adipogenic differentiation of mouse pluripotent stem cells is also significantly reduced by α-KG dimethyl ester (DMAKG), whereas osteogenic differentiation is improved [30]. α-KG can reduce the accumulation of H3k9me3 and H3K27me3 in aging bone marrow mesenchymal stem cells; reduce the occupancy of H3k9me3 and H3K27me3 in the *BMP2* and *BMP4* promoter regions and Nanog promoter regions; regulate gene methylation; increase the expression levels of the BMP signaling pathway and Nanog signaling pathway in aging bone marrow mesenchymal stem cells; and promote their migration, proliferation and osteogenic potential [31] ( Figure 2 and Table 1).

**Table:** **Table 1** The regulatory effect of α-KG on various types of bone and joint cells

Cell	Effect	Mechanism	Signaling
MSC	Inhibit adipogenic differentiation and promote osteogenic differentiation	Reduce the accumulation of H3k9me3 and H3K27me3 [30]	BMP2, BMP4
		DMAKG inhibits the adipogenic differentiation of mesenchymal stem cells [29]	–
	Inhibits the activity of stem cells	The excessive accumulation of α-KG, leading to the vacuolation of nuclear cytoplasm and chromatin condensation of mesenchymal precursor [19]	SOD2
Osteoblast	Promote osteoblast differentiation	Upregulate the expression of proline and arginine [26]	–
	Promote osteoblast differentiation	Promote the differentiation and maturation of osteoblasts and expression of Runx2 and Osterix [34]	mTOR/S6K1/S6 JNK
	Promotes osteoblast maturation and bone mineralization.	α-KG analog DMAKG activates the BMP2 pathway [29]	BMP2
	Improves the activity of osteoblasts.	α-KG participates in the metabolism of glutamate and arginine, promotes the secretion of NO by osteoblasts [38]	IDH
Osteoclast	Inhibit osteoclast differentiation and maturation	Inhibit the H3K9me3 and increase Slc7a11 expression level, produces GSH, clears ROS accumulated in mitochondria, and alleviates oxidative stress [39]	ATF4-NFATc1-RANKL, H3K9me3
		DMAKG promotes H3K27me3 demethylation inhibition and inhibits osteoclast maturation [42]	H3K27me3
		α-KG is converted to glutamine in osteoblast, and inhibits osteoclast maturation through paracrine [41]	No
Chondrocyte	α-KG alleviates the morphological abnormalities of chondrocytes	Increase the content of glutamine and hydroxyproline [44]	–
	α-KG stimulates the secretion of collagen	Increase the expression of glutamine in chondrocytes [46]	
	Promotes chondrocyte differentiation	DMOG promotes the expression and nuclear localization of HIF-1 α [49]	HIF-1α
	Promotes chondrocyte differentiation and maturation.	HIF-1α enhances hydroxyproline secretion [50]	HIF-1α
	Reduces the degeneration of nucleus pulposus cells	α-KG inhibits the p-JAK2/STAT3 pathway, reduces inflammation [51]	p-JAK2/STAT3
Synoviocyte	The decrease of α-KG under hypoxic conditions is accompanied by an increase in inflammatory factors	Under anaerobic conditions, TNF-α promote the accumulation of inflammatory factors, accompanied by the content of α-KG decreased [53]	–

### α-KG promotes osteoblast differentiation and maturation and increases new bone formation

*In vivo* studies have shown that α-KG can also alter the osteogenic activity of bone tissue by affecting collagen formation. A clinical study by Filip *et al*. [32] examined the changes in spinal bone density (BMD) in postmenopausal women after the application of α-KG. The results showed that α-KG reduced the expression level of serum C-terminal cross-linked type I collagen (CTX) and promoted the expression level of osteocalcin (OCN) in the body [32]. Moreover, with a significant increase in spinal bone density, the application of exogenous α-KG can increase osteogenic activity [32]. In another study, α-KG was converted into proline by increasing the proline residue pool through glutamic acid, which is the primary substrate for collagen synthesis. [33]. Another study suggested that α-KG can regulate osteogenic activity by modulating amino acid metabolism levels. α-KG increases the proline residue pool through glutamate and converts it into proline, thereby increasing collagen expression levels and promoting osteogenesis [26]. Researchers have established a rat model of orchiectomy to simulate tibial osteoporosis. After the application of α-KG, the bone mineral density increased, and the osteoporosis caused by the thinning of tibial cortical bone was reversed. This occurred because α-KG can significantly increase the concentration of arginine and activate the formation of osteoblasts [34] ( Figure 2).

Zurek *et al*. [35] applied α-KG to human (hFOB 1.19) and mouse (MC3T3-E1) osteoblast cell lines to verify its effect on osteogenic activity *in vitro*. The results showed that α-KG significantly activated the activity of osteoblasts, as indicated by the high expression of the Runx2 and Osterix transcription factors. The mRNA expression levels of alkaline phosphatase (ALP), type I collagen, osteopontin, and osteocalcin were also found to increase in osteoblasts [35]. Researchers have noted that the mechanism by which α-KG activates osteoblasts involves activating the JNK signaling pathway and the mTOR/S6K1/S6 signaling pathway and promoting the differentiation and maturation of osteoblasts [35]. Wang *et al*. [30] reported that α-KG can promote bone regeneration in aging mice. Another study revealed that the application of DMAKG could significantly promote bone regeneration mediated by BMP2 and improve bone mineral density in aging mice. *In vitro*, DMAKG promoted the differentiation and mineralization of osteoblasts. In addition, the inhibitory effect of lipopolysaccharide (LPS) on the differentiation of osteoblasts was reversed after the application of DMAKG [30] ( Figure 2 and Table 1).

### Osteoclast differentiation is inhibited by α-KG, which can slow bone resorption

In the bone marrow, osteoclasts are multinucleated giant cells formed by mononuclear macrophages. The functional activity of osteoclasts directly determines bone resorption activity in bone tissue, which is closely related to physiological function and inflammatory factor expression.

Osteoclast functional activity depends on the energy supply process of metabolic oxidation and decomposition, including glucose, α-KG, lactate, fatty acids, and pyruvate. Studies have shown that α-KG also plays an important role in the energy supply for bone resorption and osteoclast maturation [36]. Researchers have noted that α-KG receives the amine group on three branched-chain amino acids (BCAAs) and releases energy, which promotes osteoclast differentiation [ 37, 38]. *In vivo* studies have shown that the destruction of isocitrate dehydrogenase 2 (IDH2), which catalyzes the decarboxylation of isocitrate to α-KG, in mice is manifested by increased bone mass. *In vitro*, the decrease in α-KG content caused by *IDH2* knockout downregulated the expression level of RANKL through the ATF4-NFATc1 signaling pathway [39]. Previous studies reported that the application of DMΑKG in bone marrow-derived macrophages (BMMs) inhibited oxidative phosphorylation [ 39, 40] ( Figure 2). Researchers believe that DMAKG promotes the expression of prolyl hydroxylase 1 (PHD1) and that the transmission of the RANKL signal to the downstream inhibitor of kappa B kinase (IKK) is suppressed, ultimately suppressing osteoclast differentiation and maturation [41]. In addition, α-KG can epigenetically regulate osteoclast differentiation. These findings suggest that H3K9me binds to Slc7a11, which prevents Nrf2 from binding to the *Slc7a11* promoter region and inhibits Slc7a11 expression. However, the application of DMAKG reduced H3K9me3 and thus yielded open chromatin in Slc7a11, thereby promoting an increase in *Slc7a11* transcription levels. *Slc7a11* encodes a subunit of the cysteine/glutamate antiporter system [40]. Cysteine is produced and subsequently binds with intracellular glutamate to generate the antioxidant GSH, which clears reactive oxygen species (ROS) induced by RANKL and thus inhibits osteoclast maturation. This conclusion was validated *in vivo* and manifested as a decrease in bone resorption in mice [40]. Researchers believe that α-KG increases the level of NO in rat bones through enzymatic conversion to arginine, which increases bone mass. The increased NO in rats inhibits osteoclastic activity and promotes osteoblast activity [42]. As mentioned above, energy metabolism is closely related to gene expression. As an intermediate product of energy metabolism, α-KG regulates nucleic acid- or histone-modifying enzymes, affects related gene transcription levels, and controls the fate of cell differentiation. Another study revealed that the serine synthesis pathway (SSP) increases α-KG production through the downstream enzyme PSAT1 and ultimately promotes the differentiation and maturation of osteoclasts. The mechanism involves α-KG participating in the demethylation of H3K27me3, restoring the transcription level of osteoclasts, and increasing the expression level of NFATc1 [43] ( Figure 2 and Table 1).

### α-KG promotes cartilage development and chondrocyte maturation

**Figure 3:**
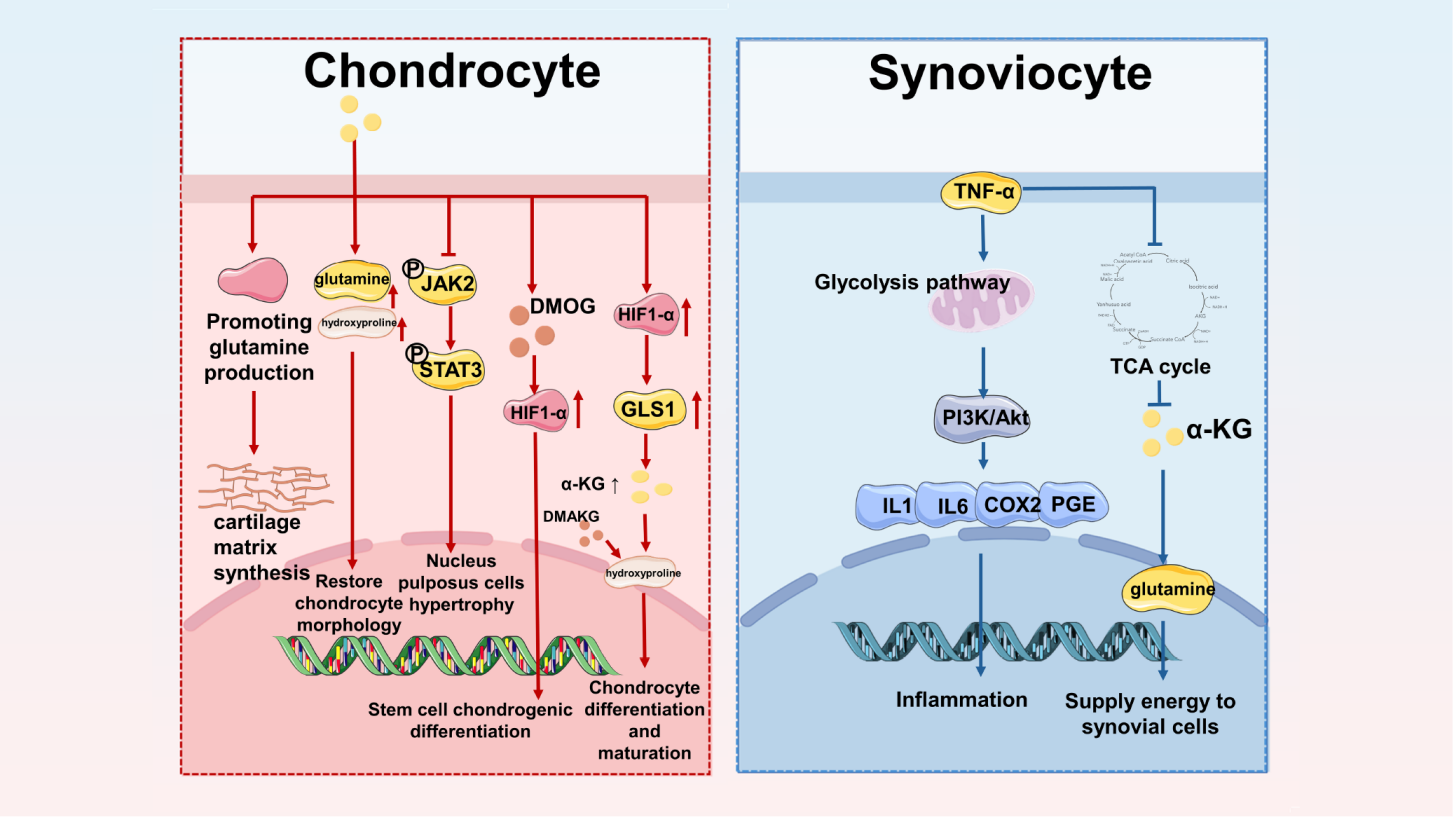
Figure 3 α-KG inhibits the inflammation of chondrocytes and synoviocytes and regulates their functional activity (Left) Chondrocytes. 1. α-KG can increase the synthesis of the extracellular matrix by increasing the expression of glutamine in chondrocytes and stimulating the secretion of collagen. 2. α-KG alleviates the morphological abnormalities of chondrocytes by stimulating the secretion of glutamine and hydroxyproline. 3. α-KG inhibits the p-JAK2/STAT3 pathway, reduces inflammation, and reduces the degeneration of nucleus pulposus cells. 4. The α-KG analogue DMOG promotes the expression and nuclear localization of hypoxia inducible factor-1 α-hydroxylase (HIF-1α) and promotes chondrocyte differentiation. 5. HIF-1α increases the content of α-KG through glutamine metabolism, enhances hydroxyproline secretion, and promotes chondrocyte differentiation and maturation. (Right) Synoviocyte. Under anaerobic conditions, TNF-α can promote the accumulation of inflammatory factors such as IL-1, IL-6, PGE and Cox through PI3K-Akt signaling pathway in glycolysis. During this process, the content of α-KG decreased but still participated in the function.

In joint tissues, α-KG also plays a regulatory role in the proliferation, differentiation, and maturation of chondrocytes. The thickness and width of the articular cartilage in male piglets and the number of chondrocytes in the superficial area of the cartilage growth plate were significantly reduced after the administration of dexamethasone. However, the application of α-KG in piglets can effectively reduce the width of the articular cartilage and growth plate cartilage, possibly because α-KG stimulates the secretion of growth hormone in the cartilage of piglets and promotes the differentiation of cartilage progenitor cells into chondrocytes [44]. Studies have shown that after gastrectomy (GX) in female rats, the number of chondrocytes in the articular cartilage decreases, and the spatial distribution of collagen fibers changes, which increases the risk of trauma [45]. Another study established that gastrectomy induced thinning of articular cartilage, while the expression of osteocalcin and osteoprotegerin in articular cartilage increased after the application of α-KG [46]. Researchers believe that α-KG provides not only an energy supply for cartilage differentiation, which is similar to the conclusions of previous studies, but also increases the content of glutamine, which is required for the synthesis of type II collagen and thus promotes the synthesis of collagen in cartilage [47]. The role of α-KG in chondrocyte differentiation, maturation, and cartilage formation was also verified in a rat model. After α-KG application, the size and shape of the chondrocytes were restored, and the spatial structure of the collagen fibers was restored through the synthesis of thin collagen, possibly because α-KG increases the content of glutamine and hydroxyproline. Another study also revealed that α-KG promoted hydroxyproline synthesis and increased the thickness of the rat tibial growth plate [ 48, 49] ( Figure 3).

Hypoxia-inducible factor-1 (HIF-1α) is generally considered to induce stem cells to differentiate into chondrocytes under hypoxia. Previous studies have shown that the application of the α-KG analogue dimethyloxalylglycine (DMOG) can induce the chondrogenic differentiation of hBM-MSCs *in vitro* and inhibit the secretion of extracellular type II and type X collagen and the formation of the extracellular matrix (ECM). DMOG was found to induce MSC cartilage differentiation by upregulating HIF-1α expression [50]. In addition, HIF-1α promotes the expression of α-KG through glutamine metabolism and stimulates an increase in hydroxyproline expression levels. DMAKG treatment increased the growth plate cartilage of the mice, which was accompanied by an increase in hydroxyproline content. We learned that α-KG may regulate the cartilage differentiation of stem cells through HIF1-α, but the function of HIF1-α can also affect the expression level of α-KG in turn. However, an increase in the α-KG concentration clearly promotes the differentiation and maturation of chondrocytes and endochondral ossification [51].

In addition, supplementation of α-KG in intervertebral disc tissue effectively improves the aging of rat nucleus pulposus (NPC) cells. In a rat model of intervertebral disc degeneration, α-KG reduces IL-1β and IL-6 levels, possibly through the inhibition of JAK2/STAT3 phosphorylation. α-KG inhibits aging-related secretory phenotypes and ECM degradation and protects the nucleus pulposus against degeneration [52] ( Figure 3 and Table 1).

### Regulation of α-KG in synoviocytes

The synovium is a special loose connective tissue covering the inner surface of a joint capsule and mainly includes macrophage-like synoviocytes, fibroblast-like synoviocytes, and a small number of dendritic-like synoviocytes [54]. Synovial fluid can reduce friction in joint movement, nourish blood vessels and nerve tissues, and keep joints in a normal physiological state. The degeneration of synovial cells is a common change in osteoarthritis (OA) and rheumatoid arthritis (RA) patients. In addition to the proliferation of synovial cells, inflammatory cell infiltration is closely related to α-ketoglutarate metabolic levels in the knee joint [53]. One study explored the relationship between fibrous synovial cells (FLSs) and α-KG metabolism under inflammatory conditions. TNF-α altered the metabolomics of FLS, reducing the metabolic flux of glucose carbon entering the TCA cycle, inhibiting the metabolic pathway of α-KG, increasing the accumulation of pyruvate in FLS, and promoting glycolysis. These results further indicate that the promotion of the glycolysis pathway by TNF-α activates the PI3K/Akt signaling pathway and triggers the expressions of IL-6, IL-8, PGE and COX-2. These results also indicate that although the content of α-KG is inhibited during this process, it can still supply energy to fibroblast synovial cells through the glutamine pathway as a carbon source [55] ( Figure 3 and Table 1). Unfortunately, the changes in α-KG in this study were secondary to the increase in TNF-α, and the study did not directly investigate whether regulating the concentration of α-KG would reduce the expression levels of inflammatory factors.

## Effects of α-KG on Anti-inflammatory and Anti-mitochondrial Oxidative Stress

### Role of α-KG in the proinflammatory response of macrophages

Hernandez *et al*. [56] injected LPS into the abdominal cavity of rats and reported that Toll-like receptors (TLRs) were activated, accompanied by increased expression of the inflammatory marker monocyte chemoattractant protein-1 (MCP-1). While the numbers of macrophages and lymphocytes also showed a marked increase, the levels of IL-1 and TNF-α were significantly elevated. During the immune response, glycolysis, a less efficient but faster functional mode, is significantly more common than the tricarboxylic acid cycle is, accompanied by a decrease in the content of intermediate products such as α-KG [56]. Changes in the concentration of α-KG are related to glycolysis under inflammatory and hypoxic conditions, and the state of the macrophage type is also affected. In terms of phenotype and function, macrophages can usually be divided into (1) the M1 phenotype, which is a typical proinflammatory phenotype, and (2) the M2 phenotype, which is an anti-inflammatory phenotype that can be refined into subgroups M2a, M2b, M2c, and M2d. Research results indicate that α-KG regulates the proportion of M1/M2 macrophages produced by macrophage polarization to regulate inflammation. On the one hand, α-KG promotes fatty acid oxidation and oxidative phosphorylation during macrophage polarization toward the M2 phenotype; on the other hand, it promotes H3K27 demethylation and initiates M2-specific marker gene promoter transcription through Jmjd3 to promote macrophage M2 polarization. In addition, a previous study suggested that α-KG inhibits the polarization of M1 macrophages by suppressing the activation of NF-κB kinase inhibitors (IKKs) [57]. In the field of bone tissue, the effect of α-KG on macrophage polarization may promote bone regeneration. Li *et al*. [58] reported that the application of α-KG in a mouse alveolar bone defect model resulted in a significant increase in the volume of new bone in the extraction socket. The author noted that the number of M1 proinflammatory macrophages was significantly reduced in the early stage, whereas in the later stage, the expressions of anti-inflammatory M2 macrophage marker genes were increased. The potential mechanism may involve α-KG increasing the number of CD206 ^+^ cells and simultaneously inhibiting IL-1β, IL-6 and TNF-α, which promote M1 polarization, and increasing the expression level of IL-4, thereby promoting M2 macrophage polarization. The regulatory effect of α-KG on macrophage polarization in the field of bone tissue may become a potential therapeutic target for the treatment of bone inflammation and bone defect diseases.

In addition to regulating the M1/M2 polarization rate of macrophages to alleviate inflammation, the content of α-KG is also related to the reprogramming of mitochondrial metabolism in inflammatory macrophages. Inflammation stimulates an increase in Nur77 receptor expression in macrophages and increases the degree of glycolysis in macrophages. Nur77-deficient macrophages are incapable of downregulating IDH expression and exhibit abnormally increased TCA cycle activity, accompanied by the accumulation of products such as α-KG and succinic acid. On the other hand, ROS and HIF-1α accumulate in the mitochondria of macrophages, leading to mitochondrial membrane hyperpolarization. *In vivo* studies revealed increased expression levels of α-KG and succinic acid, accompanied by more obvious atherosclerosis in Nur77-deficient mice [59]. In addition to the production and accumulation of inflammatory factors, the progression of inflammation is also regulated by oxidative stress. Studies have shown that electrical stimulation (ES) can increase the production of α-KG in macrophages and significantly reduce the expression level of proinflammatory cytokines and ROS production. ES increases the glucose metabolism of macrophages, promotes the TCA cycle through the pentose phosphate pathway (PPP), upregulates the expression level of α-KG, and exerts antioxidant effects by constructing an androgen receptor (CAR). The upregulation of α-KG expression and antioxidant stress by ES may become a therapeutic target for bone loss caused by abnormal activation of osteoclasts in bone tissue diseases [60].

### Relationship between α-KG and mitochondrial function and its relationship with bone lesions

The enzymes related to α-KG metabolism are mostly located in the mitochondria. A variety of metabolic pathways, including the TCA cycle, link α-KG with mitochondrial function and homeostasis. α-KG also affects mitochondrial homeostasis through energy metabolism and epigenetic regulation.

The oxidized gluconate dehydrogenase complex (OGDHc) can oxidatively phosphorylate α-KG and produce NADH and ATP. OGDHc is a key redox sensor in mitochondria, and its function affects both mitochondrial homeostasis and α-KG metabolism. In addition, domain finger protein 8 (PHF8) combined with α-KG releases H3K9me2 and H3K27me3 to bind to the promoter region, promoting the opening of the mitochondrial permeability transition pore (MPTP), accompanied by the accumulation of ROS. The redox of α-KG and its involvement in the regulation of demethylation are crucial to the function of mitochondria [ 61, 62]. The above studies revealed that the concentration of α-KG increased with the accumulation of ROS in mitochondria, but the relationship between α-KG and ROS in mitochondria differed in bone tissue. Research has shown that α-KG can clear excessive accumulation of ROS in mitochondria, and researchers believe that the mechanism involves promoting mitochondrial autophagy by supplementing α-KG, thereby reducing the accumulation of mitochondrial ROS. Furthermore, it reduces the expressions of inflammatory factors such as MMP13, IL-6, and TNF-α in cells [11].

## Relationship between α-KG and Bone Tissue-related Diseases

### α-KG increases bone mass in osteoporosis by protecting against osteogenic activity and reducing bone resorption

**Figure 4:**
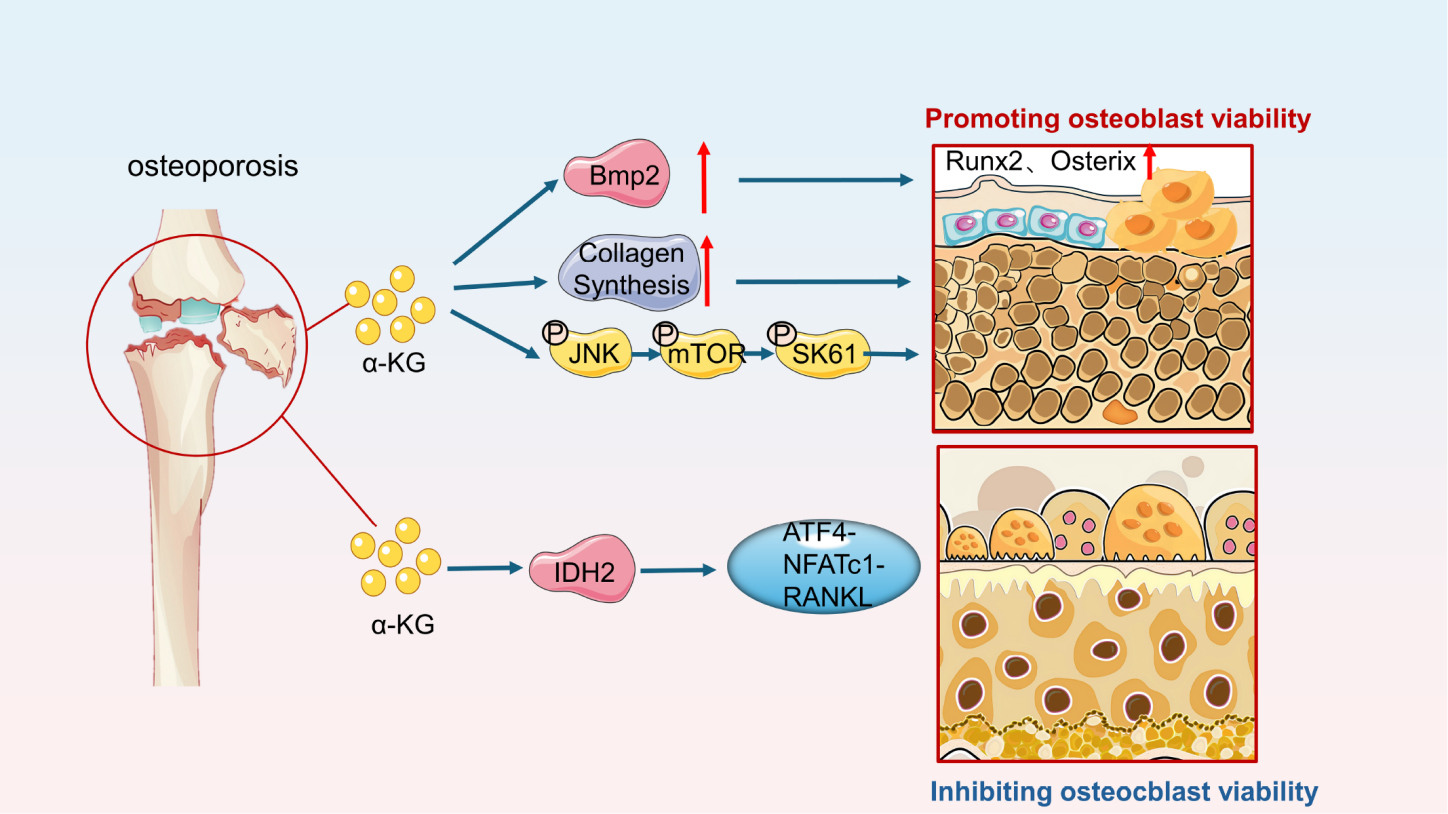
Figure 4 α-KG promotes osteogenic activity, inhibits bone resorption, and alleviates symptoms of osteoporosis α-KG increases the phosphorylation levels of JNK, mTOR, S6K1 and S6 and promotes the expression of Runx2 and Ostreix in osteoblasts. α-KG promotes the osteogenic differentiation of mesenchymal stem cells and increases the level of collagen secreted by osteoblasts. α-KG dimethyl ester (DMAKG) promotes the differentiation and mineralization of osteoblasts induced by BMP2. It can also alleviate the inflammatory response of macrophages stimulated by lipopolysaccharide (LPS) and protect against osteoblast differentiation. α-KG inhibits the activation of the Atf4-NFATc1-Rankl pathway and osteoclast activity through IDH2.

In osteoporosis, the bone microstructure and bone fragility are disrupted, resulting in low bone mass and fracture-prone bones [63]. The destruction of the bone microstructure is due to the destruction of the metabolic balance of bone and the impairment of bone marrow mesenchymal stem cell function. In addition, stem cells have the ability to undergo pluripotent differentiation to regulate osteogenic activity [64]. Many studies have shown that the application of α-KG and its related products can effectively alleviate the symptoms of osteoporosis and restore bone mass. The earliest research focused mainly on the field of animal feeding. α-KG, a key nutrient in the creole cycle, was added to the animal feed. In general, α-KG can effectively increase the bone mass of mammals such as pigs [ 65– 68] and sheep, as well as birds such as chickens [ 69– 71], and increase the degree of mineralization, cortical bone mineral density, and trabecular thickness. The application of α-KG in ovariectomized and aged rats can prevent bone loss and inhibit the development of osteoporosis [ 30, 34, 72]. Some clinical trials have shown that α-KG can effectively improve bone mass loss in postmenopausal women, which may be related to the ability of α-KG to reduce serum type I collagen C-terminal cross-linked terminal peptide (CTX) effectively [32] and regulate the content of testosterone [33]. As mentioned earlier, α-KG can activate JNK and downstream mTOR/S6K1/S6 signaling pathways to promote osteogenic differentiation, making it a potential therapeutic target for treating or preventing osteoporosis [35] ( Figure 4).

The mechanism of the action of α-KG in the treatment of osteoporosis involves four main mechanisms: (1) As an important intermediate product of energy metabolism, α-KG can provide energy for the formation of new bone and promote the deposition of collagen in the ECM. (2) α-KG promotes the activity of osteoblasts; increases the expression of osteoblast activity marker proteins such as ALP, Osterix [35] and BMP2 [30]; inhibits osteoclast differentiation and maturation; and subsequently reduces bone resorption. (3) It reduces the inflammatory response of macrophages stimulated by lipopolysaccharide (LPS) and protects against osteoblast differentiation [30]. (4) In terms of osteoclast activity, studies have shown that α-KG increases in osteoblasts and activates the cAMP pathway through IDH2, transmitting signals to the downstream NAFTc1 RANKL signaling axis, inhibiting the secretion of osteoblast-derived RANKL, and inhibiting osteoclast differentiation and maturation [ 39, 40] ( Figure 4).

### α-KG can promote the proliferation of chondrocytes, inhibit apoptosis, and reduce the inflammatory pathological progress of OA

**Figure 5:**
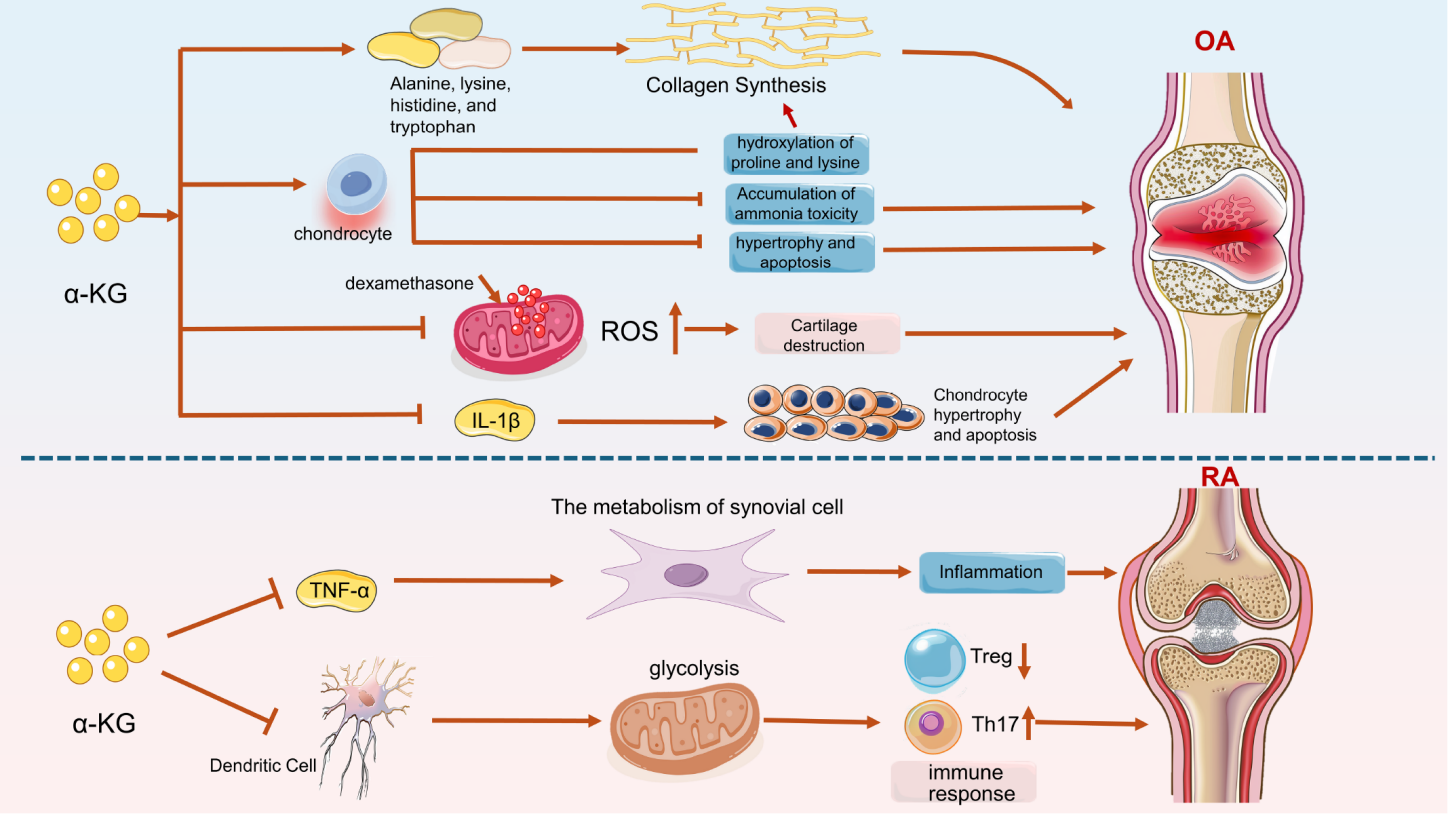
Figure 5 Regulatory role of α-KG in alleviating osteoarthritis and rheumatoid arthritis α-KG promotes the secretion of collagen in the bone and joint matrix through amino acid metabolism and alleviates bone loss in osteoarthritis. α-KG can relieve the accumulation of ammonia toxicity in chondrocytes and inhibit their apoptosis. Dexamethasone and other hormones inhibit the excessive accumulation of ROS in the mitochondria of chondrocytes and relieve oxidative stress injury in these cells. Inhibiting the expression of IL-1β in chondrocytes and inhibiting the degeneration and necrosis of inflammatory chondrocytes. α-KG inhibits the expression of TNF-α and the progression of inflammation. α-KG inhibits the energy supply in the glycolysis of dendritic cells (DCs), inhibits the expression of Th17 cells promoted by Tregs, and inhibits immune inflammation.

OA is a common chronic degenerative bone disease recognized worldwide. The main characteristics of this disease are cartilage degeneration, osteophyte development, subchondral bone sclerosis, and synovial inflammation, which together lead to pain and dysfunction of the affected joints [ 73, 74]. Through a literature review, we found that the function of chondrocytes is closely related to α-KG in both physiological and pathological states. The application of α-KG effectively inhibited the further progression of osteoarthritis. The content of α-KG in human chondrocytes induced by IL-1β decreases, whereas α-KG supplementation can reverse the inhibition of chondrocyte proliferation and reduce chondrocyte apoptosis [8]. The regulatory effect of α-KG on OA can be explained from two main aspects: glycometabolism and epigenetics ( Figure 5). *In vitro*, α-KG-fed animal experiments revealed that the concentrations of serum alanine, lysine, histidine, and tryptophan in pigs rich in α-KG were relatively high and that α-KG promoted collagen synthesis [71]. In addition, α-KG enhances proliferation and metabolic activity by reducing the ammonia toxicity produced by chondrocyte metabolism under physiological conditions [75]. Moreover, α-KG enhances the resistance of chondrocytes and their matrix protease-mediated degradation by increasing the hydroxylation of proline and lysine on collagen. It can also alleviate the hypertrophy and destruction of chondrocytes in the inflammatory state. The alleviating effect is achieved through the anti-inflammatory effect of α-KG, which is related to the regulation of mitochondrial autophagy and oxidative stress [51]. First, α-KG exerts anti-inflammatory effects by regulating mitochondrial autophagy and oxidative stress. Supplementing osteoarthritis rats with α-KG rescued degraded cartilage tissue, reversed the trend of cell apoptosis and restored chondrocyte proliferation activity in human chondrocytes cultured *in vitro* (induced by IL-1β inflammation), accompanied by increased transcription and expression of aggrecan and COL2A1 in chondrocytes [11]. In addition, the application of anti-inflammatory drugs such as dexamethasone in osteoarthritis may cause mitochondrial dysfunction in chondrocytes, accompanied by an increase in the ROS content in chondrocytes and ultimately destruction of the ECM and apoptosis of chondrocytes. α-KG effectively removes the accumulated ROS in chondrocytes and restores their function while reducing the destruction of the cartilage matrix [76]. In terms of epigenetics, researchers have noted that the levels of α-KG in human chondrocytes are reduced in OA. IL-1β and TNF-α, the two main cytokines present in arthritic joints, significantly inhibit the expression of 5-hydroxymethylcytosine (5-hmC) in chondrocytes through erasure effects and regulate DNA hydroxymethylation in chondrocytes. 5-Hmc is a completely demethylated intermediate state that regulates gene expression by combining with factors involved in the transcriptional regulation of the promoter regions of important genes involved in cartilage catabolism, such as *MMP13*, *ADAMTS-4* and *IL-1* [77] ( Figure 5). α-KG has inhibitory effects on various inflammatory factors (IL-1β, IL-6, and TNF-α). The application of α-KG to inhibit inflammatory factors and relieve the inhibition of hydroxymethylation to restore the functional defects of OA chondrocytes is a treatment option worth exploring, and further research is needed [78]. In summary, α-KG can restore the function of chondrocytes in osteoarthritis and alleviate joint tissue symptoms by regulating energy metabolism and gene methylation processes.

### α-KG can control the expression and aggregation of inflammatory-related cells and inflammatory factors in RA

The autoimmune disease rheumatoid arthritis is also an inflammatory disease characterized by a high incidence of cartilage destruction [ 79, 80]. The application of α-KG weakened the inflammatory aggregation state in a rheumatoid arthritis rat model [68]. As a common autoimmune disease, RA mainly involves synovial cells of joints, resulting in fibroblast-like changes [80]. Metabolomic analysis revealed that TNF-α in RA synovial cells altered the metabolism of fibroblast-like synovial cells, reduced the expression level of α-KG, and triggered the aggregation of other inflammatory factors (IL-1 and IL-6). Deficiency of α-KG leads to a further decrease in the anti-inflammatory ability of chondrocytes [55]. Dendritic cells (DCSs) present antigens to T cells in autoimmune diseases such as RA, leading to antigen-specific T-cell expansion or stimulating CD86 expression and promoting the differentiation and maturation of immature T cells into proinflammatory Th1 and Th17 cells. The application of particles containing α-KG to mice can alleviate the symptoms of rheumatoid arthritis associated with claws, reduce the activation of inflammatory DCs in draining lymph nodes, and activate specific anti-inflammatory regulatory T cells [81]. Another study revealed that after the application of α-KG, the proinflammatory antigen-specific T helper 17 response *in vitro* was significantly reduced, and the anti-inflammatory regulatory T response was significantly increased. Methotrexate combined with α-KG can effectively relieve symptoms in mice with rheumatoid arthritis [82] ( Figure 5). α-KG directly regulates immune cells and immune-presenting cells to act on the immune system, thereby alleviating the phenomenon of self-immune attack in the pathological process of RA.

### α-KG reduces inflammatory bone resorption in periodontal tissue and inhibits macrophage polarization

Periodontal tissue is a complex environment that includes the periodontal ligament, alveolar bone, and various types of cells and collagen molecules.

**Figure 6:**
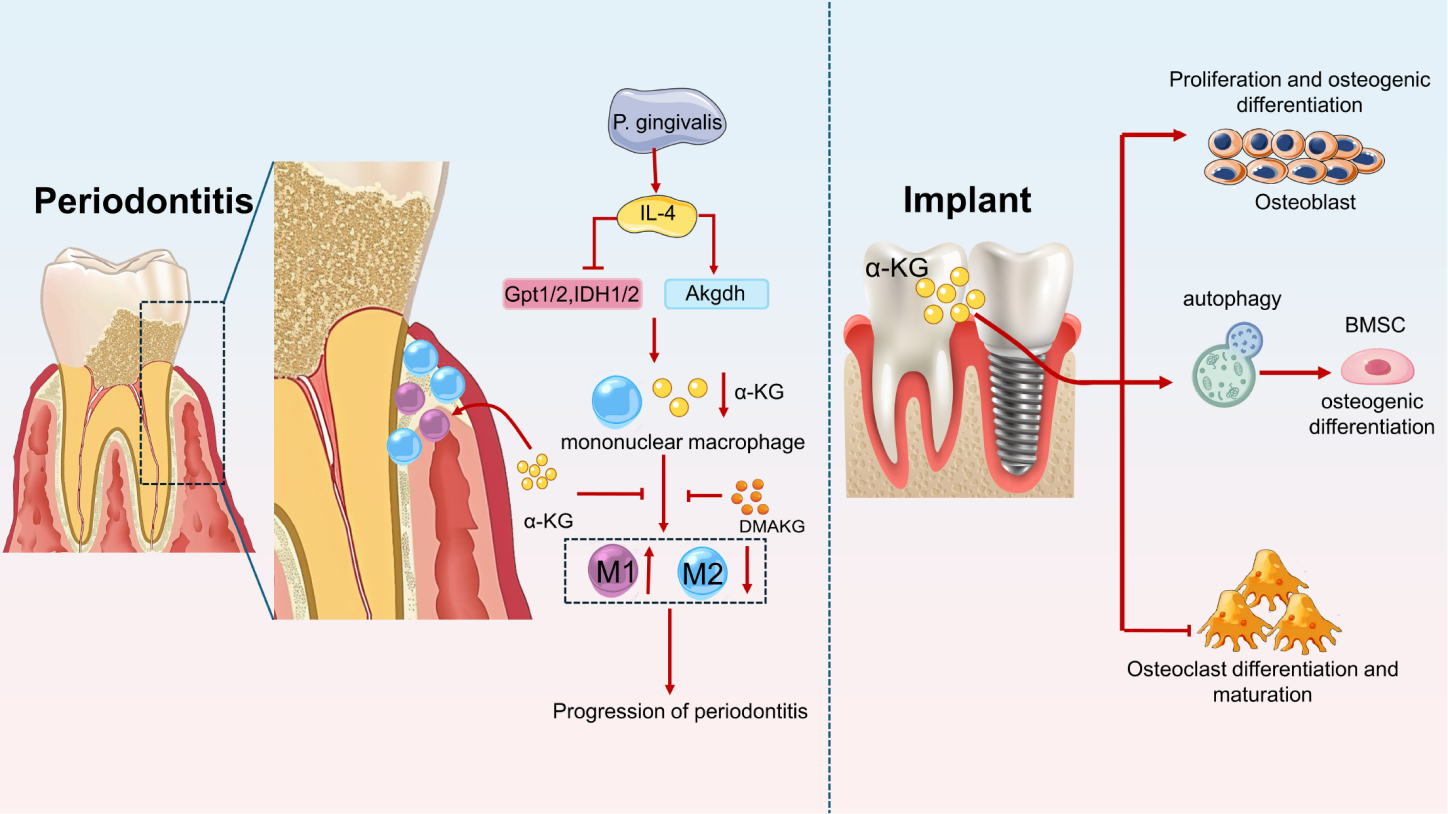
Figure 6 α-KG participates in macrophage polarization and bone metabolism in the periodontal environment α-KG promotes M2-type macrophage activation by inhibiting LPS, reduces alveolar bone absorption, and alleviates periodontal inflammation. DMAKG can inhibit the polarization of M1 macrophages in early periodontitis. α-KG around implants in alveolar bone can promote the proliferation, osteogenic differentiation and autophagy of bone marrow mesenchymal stem cells. α-KG can also promote the differentiation and maturation of osteoblasts and inhibit the differentiation and maturation of osteoclasts.

On the one hand, LPS and IL-4 expressed by *Porphyromonas gingivalis* can promote the polarization of M1 macrophages and inhibit the polarization of M2 macrophages, exacerbating periodontitis. On the other hand, *P*. *gingivalis* inhibits IDH1/2 expression and reduces α-KG expression. *P*. *gingivalis* suppresses the expressions of Gpt1/2 and IDH1/2 and increased Akgdh mRNA expression through the secretion of IL4, thereby inhibiting the expression of α-KG. Supplementation with α-KG can reverse the inhibitory effects of *P*. *gingivalis* and IL-4 on the polarization of M2 macrophages and alleviate periodontitis [83]. Another study reported that the application of α-KG could effectively reduce the activation of M1 macrophages in the early stage of defect recovery and reduce alveolar bone resorption caused by abnormal activation of osteoclasts in a mouse model of alveolar bone defects [58] ( Figure 6).

In addition to regulating the polarization direction of macrophages, α-KG can also affect the progression of periodontitis by affecting collagen synthesis and the metabolism of osteoblasts and osteoclasts. The application of α-KG can promote the healing of bone tissue around mouse alveolar bone microimplants. It promotes the proliferation and osteogenesis of mesenchymal stem cells, enhances their autophagy, and inhibits their apoptosis. α-KG can also promote osteoblast proliferation and autophagy and inhibit osteoclast differentiation and maturation. Unfortunately, researchers have not further explored the specific regulatory mechanism of α-KG in microimplant bone integration in the periodontal environment [36] ( Figure 6).

Enzymes related to α-KG in the periodontitis environment can promote collagen synthesis and alleviate periodontitis symptoms. When periodontal tissue is exposed to hypoxia, the expression levels of hydroxylase prolyl 4-hydroxylase alpha polypeptide 1 (P4HA1) and 2-oxoglutarate 5-oxygenase 2 (PLOD2) are upregulated in human gingival fibroblasts (HGFs) and human periodontal ligament cells (HPDL), accompanied by increased secretion of type I collagen. Researchers believe that excessive biting force and mechanical stress, such as trauma, can create a local hypoxic environment and induce hypoxic reactions in the periodontal ligament. The increased secretion of type I collagen can enhance collagen molecule cross-linking to protect periodontal ligaments from mechanical stress, thereby strengthening collagen fibers in the ligaments.

α-KG has been shown to participate in the synthesis of intracellular collagen, and its content decreases under hypoxic conditions due to a decrease in TCA cycle flux. It is worth exploring whether supplementation with α-KG can improve alveolar bone resorption in periodontitis and its impact on collagen secretion [ 84, 85].

### The influence of α-KG on the treatment of bone tumors

In the past 20 years, various human systems have been studied to determine the occurrence and development of tumors. Researchers have shown that ketoglutarate plays a role in many aspects related to the occurrence and development of tumors, including energy metabolism, antioxidants, and methylation-related epigenetics. Chondrosarcoma, osteosarcoma, myeloma, and giant cell tumors of bone are directly or indirectly related to the α-KG content in the body. This section mainly introduces the regulatory effect of α-KG in bone tissue tumors.

#### Chondrosarcoma

Chondrosarcoma is a common malignant bone tumor. Chondrosarcoma is a heterogeneous primary bone malignant tumor that can produce a hyaline cartilage matrix. It can occur in the medullary cavity or periosteum. IDH is a key enzyme in the TCA cycle and is responsible for converting isocitrate to α-KG. Mutations in the *IDH* gene in chondrocytes can induce the occurrence of chondrosarcoma in mice. IDH1-R132Q mutations can catalyze the conversion of α-ketoglutarate to d-2-hydroxyglutarate (d-2HG) in chondrocytes, leading to increased proliferation and gene expression in hypertrophic chondrocytes, ultimately leading to the development of multiple endogenous chondroma-like lesions [86].

Nakagawa *et al*. [87] suggested that the activity of chondrosarcoma cells decreases after stimulation with IDH inhibitors. The mechanism involves the upregulation of *Sox9* and *CDKN1C* gene expressions via the regulation of histone H3K9me3 demethylation, which causes cell cycle arrest in chondrocytes. In addition, Zhang *et al*. [88] reported that glutamine-derived α-KG promoted the differentiation of hypertrophic chondrocytes and regulated the proliferation of chondrocytes during the development of chondroma-like lesions in mice. On the other hand, glutamine-derived nonessential amino acids play important roles in preventing cell apoptosis. Researchers have proposed that α-KG supplementation may be a therapeutic method to inhibit the growth of chondroma and chondrosarcoma.

#### Osteosarcoma

Osteosarcoma is the most common primary bone malignancy and is still a disease with high mortality in children and adolescents. In addition to being a central metabolic regulator of tumor fate, α-KG plays a vital role in tumor occurrence and progression. By stimulating osteosarcoma cells with α-KG *in vitro*, α-KG inhibited the proliferation of osteosarcoma cell lines in a concentration-dependent manner and blocked the proliferation of osteosarcoma cells in the G1 phase. Moreover, α-KG can induce osteosarcoma cell apoptosis through the activation of c-Jun N-terminal protein kinase (JNK) [89]. In addition to its direct regulatory effect on osteosarcoma cells, α-KG can reduce the activation of extracellular signal-regulated kinase (ERK1/2) in osteosarcoma tissues, significantly inhibiting the migration and invasion of cancer cells *in vitro*. In addition, α-KG reduces the production of transforming growth factor-α (TGF-α) and angiogenic vascular endothelial growth factor (VEGF), which can reduce the generation of vascular tissue in osteosarcoma [89]. In conclusion, α-KG can affect the occurrence and development of osteosarcoma.

#### Giant cell tumor of bone

Twenty percent of bone tumors in Asia are giant cell tumors of bone (GCTB), most of which are benign [90]. The GCTB is characterized by a high number of multinucleated osteoclasts, including myeloid monocytes and mesenchymal fibroblasts such as giant cells [91]. IDH1/2 mutations can convert α-KG into R (-)-2-hydroxyglutaric acid, which is a cancer metabolite. However, no studies have attempted to use α-KG and its products to alleviate giant cell tumors of bone.

As elucidated in this section, α-KG serves as a crucial metabolic intermediate and cofactor for several epigenetic regulatory enzymes that play roles in DNA demethylation, transcription, and epigenetic modifications. The potential clinical applications of α-KG have consistently been a focal point of research interest. It has been demonstrated that α-KG is absorbed through the intestinal epithelium in mammals and is frequently administered orally in human subjects. Initial investigations into the role of α-KG as a source of cellular energy metabolism were conducted in both animal models [92] and human studies [93]. Rejuvants, complexes containing α-KG and vitamins, showed an average reduction of 8 years in biological aging after 7 months of application [94], which can effectively delay aging through methylation. α-KG has also been shown to delay muscle atrophy in the human body by inhibiting the interaction between β2-adrenergic receptors and prolyl hydroxylase3, thereby suppressing muscle breakdown [95]. On the other hand, it promotes muscle protein synthesis and prevents muscle protein breakdown by reducing the concentration of free glutamine. Moreover, myocardial cells are protected by α-KG. Adding 28 grams of α-KG to blood cardiac arrest fluid during human cardiac surgery can increase myocardial oxygen extraction, reduce the production of myocardial lactate in blood cardiac arrest fluid, and alleviate ischemic injury [ 96, 97]. In addition, the clinical application of α-KG is related to the balance and metabolic regulation of the gut microbiota [98] and anti-tumor therapy [ 99, 100].

In the context of osteoporosis treatment, research conducted on postmenopausal women with this condition demonstrated that the administration of daily oral calcium α-ketoglutarate (6 g α-KG over a duration of six months) resulted in a 1.6% increase in bone density relative to baseline measurements [32]. The underlying mechanism involves the regulation of epigenetic processes by α-KG, which aligns with previously discussed regulatory effects of α-KG on osteoblast activity. Furthermore, as previously noted, the protective effects of α-KG on osteogenic activity and cartilage integrity under inflammatory conditions, along with its inhibitory impact on osteoclast-mediated bone resorption, indicate its potential therapeutic role in human inflammatory bone destruction diseases such as OA and RA [ 81, 101].

Although animal studies have demonstrated that exogenous α-KG can effectively mitigate inflammation and restore bone mass in animal models of OA and RA, it is unfortunate that no clinical trials have yet been conducted to explore its application in humans. Notably, some studies have shown that the absorption efficiency of oral exogenous α-KG in the intestinal epithelium gradually decreases with increasing age [ 102, 103]. In patients with bone tissue diseases, which have a high incidence rate among middle-aged and elderly individuals, it is essential to consider the form of α-KG that optimizes absorption. This aspect warrants further investigation.

## Conclusion

At the cellular level, α-KG promotes the osteogenic differentiation of stem cells, increases the activity of osteoblasts to promote osteogenesis, and inhibits the bone resorption activity of osteoclasts. α-KG in articular cartilage promotes the differentiation and maturation of chondrocytes and the formation of a cartilage matrix. The protective effect of α-KG on bone has practical value in the treatment of abnormal bone loss symptoms in various bone tissue diseases (osteoporosis, osteoarthritis, *etc*.). α-KG was initially used in animal feed to increase bone tissue density and relieve osteoporosis symptoms. The application of α-KG in a rat model of osteoarthritis has also been shown to alleviate the progression of inflammation. As a potential therapeutic target for osteoporosis, osteoarthritis and other diseases, α-KG needs more clinical trials to develop effective targeted drugs.

## References

[1] Harrison AP, Pierzynowski SC. Biological effects of 2-oxoglutarate with particular emphasis on the regulation of protein, mineral and lipid absorption/metabolism, muscle performance, kidney function, bone formation and cancerogenesis, all viewed from a healthy ageing perspective state of the art-review article.
JPhysiolPharmacol 2008, 59: 91–106. https://pubmed.ncbi.nlm.nih.gov/18802218/.

[2] Dąbek M, Kruszewska D, Filip R, Hotowy A, Pierzynowski Ł, Wojtasz-Pająk A, Szymanczyk S (2005). *α*-Ketoglutarate (AKG) absorption from pig intestine and plasma pharmacokinetics. Anim Physiol Nutr.

[3] Ng KT, O′Dowd BS, Rickard NS, Robinson SR, Gibbs ME, Rainey C, Zhao WQ (1997). Complex roles of glutamate in the Gibbs-Ng model of one-trial aversive learning in the new-born chick. Neurosci BioBehav Rev.

[4] Lambert BD, Stoll B, Niinikoski H, Burrin DG, Pierzynowski S (2002). Net portal absorption of enterally fed α-ketoglutarate is limited in young pigs. J Nutr.

[5] Lambert BD, Filip R, Stoll B, Junghans P, Derno M, Hennig U, Souffrant WB (2006). First-pass metabolism limits the intestinal absorption of enteral α-ketoglutarate in young pigs. J Nutr.

[6] Forni MF, Peloggia J, Trudeau K, Shirihai O, Kowaltowski AJ (2016). Murine mesenchymal stem cell commitment to differentiation is regulated by mitochondrial dynamics. Stem Cells.

[7] Li Q, Gao Z, Chen Y, Guan MX (2017). The role of mitochondria in osteogenic, adipogenic and chondrogenic differentiation of mesenchymal stem cells. Protein Cell.

[8] C. Zanker, K. Hind. The effect of energy balance on endocrine function and bone health in youth.
*
Med Sport Sci
* 2007, 51: 81-101. https://doi.org/10.1159/000103006.

[9] Lee HJ, Jung YH, Choi GE, Kim JS, Chae CW, Lim JR, Kim SY (2019). O-cyclic phytosphingosine-1-phosphate stimulates HIF1α-dependent glycolytic reprogramming to enhance the therapeutic potential of mesenchymal stem cells. Cell Death Dis.

[10] Martinez VG, Ontoria-Oviedo I, Ricardo CP, Harding SE, Sacedon R, Varas A, Zapata A (2017). Overexpression of hypoxia-inducible factor 1 alpha improves immunomodulation by dental mesenchymal stem cells. Stem Cell Res Ther.

[11] Liu L, Zhang W, Liu T, Tan Y, Chen C, Zhao J, Geng H (2023). The physiological metabolite α-ketoglutarate ameliorates osteoarthritis by regulating mitophagy and oxidative stress. Redox Biol.

[12] X. Zhang, G.F. Jia. RNA epigenetic modification: N6-methyladenosine.
*
Yi Chuan
* 2016, 38: 275–288. https://doi.org/10.16288/j.yczz.16-049.

[13] Yi SJ, Lee H, Lee J, Lee K, Kim J, Kim Y, Park JI (2019). Bone remodeling: histone modifications as fate determinants of bone cell differentiation. Int J Mol Sci.

[14] Naeini SH, Mavaddatiyan L, Kalkhoran ZR, Taherkhani S, Talkhabi M (2023). Alpha-ketoglutarate as a potent regulator for lifespan and healthspan: Evidences and perspectives. Exp Gerontology.

[15] Liu S, He L, Yao K (2018). The antioxidative function of alpha-ketoglutarate and its applications. Biomed Res Int.

[16] Asadi Shahmirzadi A, Edgar D, Liao CY, Hsu YM, Lucanic M, Asadi Shahmirzadi A, Wiley CD (2020). Alpha-ketoglutarate, an endogenous metabolite, extends lifespan and compresses morbidity in aging mice. Cell Metab.

[17] Zhang Z, He C, Gao Y, Zhang L, Song Y, Zhu T, Zhu K (2021). α-ketoglutarate delays age‐related fertility decline in mammals. Aging Cell.

[18] Su Y, Wang T, Wu N, Li D, Fan X, Xu Z, Mishra SK (2019). Alpha-ketoglutarate extends Drosophila lifespan by inhibiting mTOR and activating AMPK. Aging.

[19] Chai M, Jiang M, Vergnes L, Fu X, de Barros SC, Doan NB, Huang W (2019). Stimulation of hair growth by small molecules that activate autophagy. Cell Rep.

[20] Singh K, Krug L, Basu A, Meyer P, Treiber N, Vander Beken S, Wlaschek M (2017). Alpha-ketoglutarate curbs differentiation and induces cell death in mesenchymal stromal precursors with mitochondrial dysfunction. Stem Cells.

[21] Hou Y (2011). Alpha-Ketoglutarate and intestinal function. Front Biosci.

[22] Portais JC, Voisin P, Merle M, Canioni P (1996). Glucose and glutamine metabolism in C6 glioma cells studied by carbon 13 NMR. Biochimie.

[23] Luo Q, Qian R, Qiu Z, Yamamoto FY, Du Y, Lin X, Zhao J (2023). Dietary α-ketoglutarate alleviates glycinin and β-conglycinin induced damage in the intestine of mirror carp (
*Cyprinus carpio*). Front Immunol.

[24] Wu N, Yang M, Gaur U, Xu H, Yao Y, Li D (2016). Alpha-ketoglutarate: physiological functions and applications. Biomolecules Ther.

[25] Velvizhi S, Dakshayani KB, Subramanian P (2002). Effects of α-ketoglutarate on antioxidants and lipid peroxidation products in rats treated with ammonium acetate. Nutrition.

[26] Tapiero H, Mathé G, Couvreur P, Tew KD (2002). II. Glutamine and glutamate. Biomed Pharmacother.

[27] Lamande SR, Bateman JF (1999). Procollagen folding and assembly: the role of endoplasmic reticulum enzymes and molecular chaperones. Semin Cell Dev Biol.

[28] Tian J, Yang F, Bao X, Jiang Q, Li Y, Yao K, Yin Y (2023). Dietary alpha-ketoglutarate supplementation improves bone growth, phosphorus digestion, and growth performance in piglets. Animals.

[29] Liu K, Wu Y, Yang W, Li T, Wang Z, Xiao S, Peng Z (2024). α-Ketoglutarate improves ovarian reserve function in primary ovarian insufficiency by inhibiting NLRP3-mediated pyroptosis of granulosa cells. Mol Nutr Food Res.

[30] Wang Z, Hu J, Faber J, Miszuk J, Sun H (2022). Locally delivered metabolite derivative promotes bone regeneration in aged mice. ACS Appl Bio Mater.

[31] Wang Y, Deng P, Liu Y, Wu Y, Chen Y, Guo Y, Zhang S (2020). Alpha-ketoglutarate ameliorates age-related osteoporosis via regulating histone methylations. Nat Commun.

[32] Filip RS, Pierzynowski SG, Lindegard B, Wernerman J, Haratym-Maj A, Podgurniak M (2007). Alpha-ketoglutarate decreases serum levels of c-terminal cross-linking telopeptide of type i collagen (CTX) in postmenopausal women with osteopenia: six-month study. Int J Vitamin Nutr Res.

[33] Filip R, Raszewski G (2009). Bone mineral density and bone turnover in relation to serum leptin, α‐ketoglutarate and sex steroids in overweight and obese postmenopausal women. Clin Endocrinol.

[34] Radzki RP, Bienko M, Pierzynowski SG (2012). Anti-osteopenic effect of alpha-ketoglutarate sodium salt in ovariectomized rats. J Bone Miner Metab.

[35] Żurek A, Mizerska-Kowalska M, Sławińska-Brych A, Kaławaj K, Bojarska-Junak A, Kandefer-Szerszeń M, Zdzisińska B (2019). Alpha ketoglutarate exerts a pro-osteogenic effect in osteoblast cell lines through activation of JNK and mTOR/S6K1/S6 signaling pathways. Toxicol Appl Pharmacol.

[36] Liu R, Gao Y, Huang L, Shi B, Yin X, Zou S (2023). Alpha‐ketoglutarate up‐regulates autophagic activity in peri‐implant environment and enhances dental implant osseointegration in osteoporotic mice. J Clinic Periodontol.

[37] Williams JP, Blair HC, McDonald JM, McKenna MA, Jordan SE, Williford J, Hardy RW (1997). Regulation of osteoclastic bone resorption by glucose. Biochem Biophys Res Commun.

[38] Go M, Shin E, Jang SY, Nam M, Hwang GS, Lee SY (2022). BCAT1 promotes osteoclast maturation by regulating branched-chain amino acid metabolism. Exp Mol Med.

[39] Lee SH, Lee SH, Lee JH, Park JW, Kim JE (2019). IDH2 deficiency increases bone mass with reduced osteoclastogenesis by limiting RANKL expression in osteoblasts. Bone.

[40] Lee S, Kim HS, Kim MJ, Min KY, Choi WS, You JS (2021). Glutamine metabolite α-ketoglutarate acts as an epigenetic co-factor to interfere with osteoclast differentiation. Bone.

[41] Tian J, Bao X, Yang F, Tang X, Jiang Q, Li Y, Yao K (2023). Elevation of intracellular alpha-ketoglutarate levels inhibits osteoclastogenesis by suppressing the NF-κB signaling pathway in a PHD1-dependent manner. Nutrients.

[42] Radzki RP, Bieńko M, Filip R, Pierzynowski SG (2016). The protective and therapeutic effect of exclusive and combined treatment with alpha-ketoglutarate sodium salt and ipriflavone on bone loss in orchidectomized rats. J Nutr Health Aging.

[43] Stegen S, Moermans K, Stockmans I, Thienpont B, Carmeliet G (2024). The serine synthesis pathway drives osteoclast differentiation through epigenetic regulation of NFATc1 expression. Nat Metab.

[44] E. Tomaszewska, P. Dobrowolski, J. Wydrych. Postnatal administration of 2-oxoglutaric acid improves articular and growth plate cartilages and bone tissue morphology in pigs prenatally treated with dexamethasone
*
J Physiol Pharmacol
* 2012, 6: 547–554. https://pubmed.ncbi.nlm.nih.gov/23211309/.

[45] Dobrowolski P, Tomaszewska E, Muszyński S, Blicharski T, Pierzynowski SG (2017). Dietary 2-oxoglutarate prevents bone loss caused by neonatal treatment with maximal dexamethasone dose. Exp Biol Med (Maywood).

[46] Dobrowolski PJ, Piersiak T, Surve VV, Kruszewska D, Gawron A, Pacuska P, Håkanson R (2008). Dietary α-ketoglutarate reduces gastrectomy-evoked loss of calvaria and trabecular bone in female rats. Scand J Gastroenterol.

[47] Tomaszewska E, Dobrowolski P, Prost Ł, Hułas-stasiak M, Muszyński S, Blicharski T (2016). The effect of supplementation of a glutamine precursor on the growth plate, articular cartilage and cancellous bone in fundectomy-induced osteopenic bone. J Vet Med Sci.

[48] Dobrowolski P, Tomaszewska E, Kurlak P, Pierzynowski SG (2016). Dietary 2-oxoglutarate mitigates gastrectomy-evoked structural changes in cartilage of female rats. Exp Biol Med (Maywood).

[49] Dobrowolski P, Tomaszewska E, Bienko M, Radzki RP, Pierzynowski SG (2013). The effect of dietary administration of 2-oxoglutaric acid on the cartilage and bone of growing rats. Br J Nutr.

[50] Taheem DK, Foyt DA, Loaiza S, Ferreira SA, Ilic D, Auner HW, Grigoriadis AE (2018). Differential regulation of human bone marrow mesenchymal stromal cell chondrogenesis by hypoxia inducible factor-1α hydroxylase inhibitors. Stem Cells.

[51] Stegen S, Laperre K, Eelen G, Rinaldi G, Fraisl P, Torrekens S, Van Looveren R (2019). HIF-1α metabolically controls collagen synthesis and modification in chondrocytes. Nature.

[52] Xu HW, Fang XY, Liu XW, Zhang SB, Yi YY, Chang SJ, Chen H (2023). α-Ketoglutaric acid ameliorates intervertebral disk degeneration by blocking the IL-6/JAK2/STAT3 pathway. Am J Physiol Cell Physiol.

[53] Jiang H, Liu J, Qin XJ, Chen YY, Gao JR, Meng M, Wang Y (2018). Gas chromatography‑time of flight/mass spectrometry‑based metabonomics of changes in the urinary metabolic profile in osteoarthritic rats. Exp Ther Med.

[54] Schett G, Tohidast-Akrad M, Steiner G, Smolen J (2001). The stressed synovium. Arthritis Res Ther.

[55] Manosalva C, Alarcon P, Quiroga J, Teuber S, Carretta MD, Bustamante H, Lopez-Muñoz R (2023). Bovine tumor necrosis factor-alpha Increases IL-6, IL-8, and PGE2 in bovine fibroblast-like synoviocytes by metabolic reprogramming. Sci Rep.

[56] Hernandez-Baixauli J, Abasolo N, Palacios-Jordan H, Foguet-Romero E, Suñol D, Galofré M, Caimari A (2022). Imbalances in TCA, short fatty acids and one-carbon metabolisms as important features of homeostatic disruption evidenced by a multi-omics integrative approach of LPS-induced chronic inflammation in male wistar rats. Int J Mol Sci.

[57] Ren W, Xia Y, Chen S, Wu G, Bazer FW, Zhou B, Tan B (2019). Glutamine metabolism in macrophages: a novel target for obesity/type 2 diabetes. Adv Nutr.

[58] Li Y, Liu L, Li Y, Song W, Shao B, Li H, Lin W (2023). Alpha-ketoglutarate promotes alveolar bone regeneration by modulating M2 macrophage polarization. Bone Rep.

[59] Koenis DS, Medzikovic L, van Loenen PB, van Weeghel M, Huveneers S, Vos M, Evers-van Gogh IJ (2018). Nuclear receptor nur77 limits the macrophage inflammatory response through transcriptional reprogramming of mitochondrial metabolism. Cell Rep.

[60] Uemura M, Maeshige N, Yamaguchi A, Ma X, Matsuda M, Nishimura Y, Hasunuma T (2023). Electrical stimulation facilitates NADPH production in pentose phosphate pathway and exerts an anti-inflammatory effect in macrophages. Sci Rep.

[61] Ying Z, Xiang G, Zheng L, Tang H, Duan L, Lin X, Zhao Q (2018). Short-term mitochondrial permeability transition pore opening modulates histone lysine methylation at the early phase of somatic cell reprogramming. Cell Metab.

[62] Chang LC, Chiang SK, Chen SE, Hung MC. Targeting 2-oxoglutarate dehydrogenase for cancer treatment.
*
Am J Cancer Res
* 2022, 12: 1436–1455. https://pubmed.ncbi.nlm.nih.gov/35530286/.

[63] Aspray TJ, Hill TR. Osteoporosis and the ageing skeleton.
*
Subcell Biochem
* 2019, 91: 453–476. https://doi.org/10.1007/978-981-13-3681-2_16.

[64] Srivastava M, Deal C (2002). Osteoporosis in elderly: prevention and treatment. Clin Geriatric Med.

[65] Harrison AP, Tygesen MP, Sawa-Wojtanowicz B, Husted S, Tatara MR (2004). α-Ketoglutarate treatment early in postnatal life improves bone density in lambs at slaughter. Bone.

[66] Tatara MR, Śliwa E, Krupski W, Brodzki A, Pasternak K (2006). Ornithine alpha-ketoglutarate increases mineralization and mechanical properties of tibia in turkeys. Bone.

[67] Andersen NK, Tatara MR, Krupski W, Majcher P, Harrison AP (2008). The long-term effect of α-ketoglutarate, given early in postnatal life, on both growth and various bone parameters in pigs. Anim Physiol Nutr.

[68] Tatara MR, Krupski W, Tymczyna B, Studziński T (2012). Effects of combined maternal administration with alpha-ketoglutarate (AKG) and β-hydroxy-β-methylbutyrate (HMB) on prenatal programming of skeletal properties in the offspring. Nutr Metab (Lond).

[69] Tatara MR, Brodzki A, Krupski W, śliwa E, Silmanowicz P, Majcher P, Pierzynowski SG (2005). Effects of alpha-ketoglutarate on bone homeostasis and plasma amino acids in turkeys. Poultry Sci.

[70] Tomaszewska E, Świątkiewicz S, Arczewska-Włosek A, Wojtysiak D, Dobrowolski P, Domaradzki P, Świetlicka I,
*et al*. Alpha-ketoglutarate: an effective feed supplement in improving bone metabolism and muscle quality of laying hens: a preliminary study.
*
Animals
* 2020, 10: 2420. https://doi.org/10.3390/ani10122420.

[71] Tomaszewska E, Burmańczuk N, Dobrowolski P, Świątkiewicz M, Donaldson J, Burmańczuk A, Mielnik-Błaszczak M,
*et al.* The protective role of alpha-ketoglutaric acid on the growth and bone development of experimentally induced perinatal growth-retarded piglets.
*
Animals
* 2021, 11: 137. https://doi.org/10.3390/ani11010137.

[72] Cheng C, Xing Z, Hu Q, Kong N, Liao C, Xu S, Zhang J (2024). A bone-targeting near-infrared luminescence nanocarrier facilitates alpha-ketoglutarate efficacy enhancement for osteoporosis therapy. Acta BioMater.

[73] Xia B, Di Chen B, Zhang J, Hu S, Jin H, Tong P (2014). Osteoarthritis pathogenesis: a review of molecular mechanisms. Calcif Tissue Int.

[74] Abramoff B, Caldera FE (2020). Caldera, osteoarthritis: pathology, diagnosis, and treatment options. Med Clin N Am.

[75] Singh D, Vishnoi T, Kumar A (2013). Effect of alpha-ketoglutarate on growth and metabolism of cells cultured on three-dimensional cryogel matrix. Int J Biol Sci.

[76] Li Q, Chen H, Li Z, Zhang F, Chen L (2022). Glucocorticoid caused lactic acid accumulation and damage in human chondrocytes via ROS-mediated inhibition of monocarboxylate transporter 4. Bone.

[77] Urita A, Matsuhashi T, Onodera T, Nakagawa H, Hato M, Amano M, Seito N (2011). Alterations of high-mannose type
*N*-glycosylation in human and mouse osteoarthritis cartilage. Arthritis Rheumatism.

[78] Haseeb A, Makki MS, Haqqi TM (2014). Modulation of ten-eleven translocation 1 (TET1), isocitrate dehydrogenase (IDH) expression, α-ketoglutarate (α-KG), and DNA hydroxymethylation levels by interleukin-1β in primary human chondrocytes. J Biol Chem.

[79] McInnes IB, Schett G (2011). The pathogenesis of rheumatoid arthritis. N Engl J Med.

[80] Wasserman AM. Diagnosis and management of rheumatoid arthritis.
*
Am Fam Physician
* 2011, 84: 1245–1252. https://pubmed.ncbi.nlm.nih.gov/22150658/.

[81] Mangal JL, Inamdar S, Le T, Shi X, Curtis M, Gu H, Acharya AP (2021). Inhibition of glycolysis in the presence of antigen generates suppressive antigen-specific responses and restrains rheumatoid arthritis in mice. Biomaterials.

[82] Mangal JL, Inamdar S, Suresh AP, Jaggarapu MMCS, Esrafili A, Ng ND, Acharya AP (2022). Short term, low dose alpha-ketoglutarate based polymeric nanoparticles with methotrexate reverse rheumatoid arthritis symptoms in mice and modulate T helper cell responses. BioMater Sci.

[83] Yu S, Ding L, Liang D, Luo L (2018). *Porphyromonas gingivalis* inhibits M2 activation of macrophages by suppressing α‐ketoglutarate production in mice. Mol Oral Microbiol.

[84] Morimoto C, Takedachi M, Kawasaki K, Shimomura J, Murata M, Hirai A, Kawakami K (2021). Hypoxia stimulates collagen hydroxylation in gingival fibroblasts and periodontal ligament cells. J Periodontol.

[85] Balci Yuce H, Karatas Ö, Tulu F, Altan A, Gevrek F (2019). Effect of diabetes on collagen metabolism and hypoxia in human gingival tissue: a stereological, histopathological, and immunohistochemical study. Biotechnic Histochem.

[86] Hirata M, Sasaki M, Cairns RA, Inoue S, Puviindran V, Li WY, Snow BE (2015). Mutant
*IDH* is sufficient to initiate enchondromatosis in mice. Proc Natl Acad Sci USA.

[87] Nakagawa M, Nakatani F, Matsunaga H, Seki T, Endo M, Ogawara Y, Machida Y (2019). Selective inhibition of mutant IDH1 by DS-1001b ameliorates aberrant histone modifications and impairs tumor activity in chondrosarcoma. Oncogene.

[88] Zhang H, Puviindran V, Nadesan P, Ding X, Shen L, Tang YJ, Tsushima H (2022). Distinct roles of glutamine metabolism in benign and malignant cartilage tumors with IDH mutations. J Bone Miner Res.

[89] Kaławaj K, Sławińska-Brych A, Mizerska-Kowalska M, Żurek A, Bojarska-Junak A, Kandefer-Szerszeń M, Zdzisińska B (2020). Alpha ketoglutarate exerts
*in vitro* anti-osteosarcoma effects through inhibition of cell proliferation, induction of apoptosis via the jnk and caspase 9-dependent mechanism, and suppression of TGF-β and VEGF production and metastatic potential of cells. Int J Mol Sci.

[90] Mendenhall WM, Zlotecki RA, Scarborough MT, Gibbs CP, Mendenhall NP (2006). Giant cell tumor of bone. Am J Clin Oncol.

[91] Nagano A, Urakawa H, Tanaka K, Ozaki T (2022). Current management of giant-cell tumor of bone in the denosumab era. Jpnese J Clin Oncol.

[92] Wang L, Hou Y, Yi D, Li Y, Ding B, Zhu H, Liu J (2015). Dietary supplementation with glutamate precursor α-ketoglutarate attenuates lipopolysaccharide-induced liver injury in young pigs. Amino Acids.

[93] Donati L, Ziegler F, Pongelli G, Signorini M (1999). Nutritional and clinical efficacy of ornithine alphaketog-ketoglutaratein severe burn patients. Clin Nutr.

[94] Demidenko O, Barardo D, Budovskii V, Finnemore R, Palmer Iii FR, Kennedy BK, Budovskaya YV (2021). Rejuvant®, a potential life-extending compound formulation with alpha-ketoglutarate and vitamins, conferred an average 8 year reduction in biological aging, after an average of 7 months of use, in the TruAge DNA methylation test. Aging.

[95] Cai X, Yuan Y, Liao Z, Xing K, Zhu C, Xu Y, Yu L (2018). α‐Ketoglutarate prevents skeletal muscle protein degradation and muscle atrophy through PHD3/ADRB2 pathway. FASEB J.

[96] Kjellman U, Björk K, Ekroth R, Karlsson H, Nilsson F, Svensson G, Jagenburg R (1995). α-Ketoglutarate for myocardial protection in heart surgery. Lancet.

[97] Kjellman UW, Björk K, Ekroth R, Karlsson H, Jagenburg R, Nilsson FN, Svensson G (1997). Addition of α-ketoglutarate to blood cardioplegia improves cardioprotection. Ann Thoracic Surg.

[98] Chen S, Bin P, Ren W, Gao W, Liu G, Yin J, Duan J (2017). Alpha-ketoglutarate (AKG) lowers body weight and affects intestinal innate immunity through influencing intestinal microbiota. Oncotarget.

[99] Zdzisińska B, Żurek A, Kandefer-Szerszeń M (2017). Alpha-ketoglutarate as a molecule with pleiotropic activity: well-known and novel possibilities of therapeutic use. Arch Immunol Ther Exp.

[100] Raimundo N, Baysal BE, Shadel GS (2011). Revisiting the TCA cycle: signaling to tumor formation. Trends Mol Med.

[101] Valverde-Franco G, Hum D, Matsuo K, Lussier B, Pelletier JP, Fahmi H, Kapoor M,
*et al*. The
*in vivo* effect of prophylactic subchondral bone protection of osteoarthritic synovial membrane in bone-specific Ephb4-overexpressing mice.
Am J Pathol 2015, 185: 335–646. https://doi.org/10.1016/j.ajpath.2014.10.004.

[102] Tian Q, Zhao J, Yang Q, Wang B, Deavila JM, Zhu MJ, Du M (2020). Dietary alpha-ketoglutarate promotes beige adipogenesis and prevents obesity in middle-aged mice. Aging Cell.

[103] Woudstra T, Thomson ABR (2002). Nutrient absorption and intestinal adaptation with ageing. Best Pract Res Clin Gastroenterol.

